# Alternative transcription start sites contribute to acute-stress-induced transcriptome response in human skeletal muscle

**DOI:** 10.1186/s40246-022-00399-8

**Published:** 2022-07-22

**Authors:** Pavel A. Makhnovskii, Oleg A. Gusev, Roman O. Bokov, Guzel R. Gazizova, Tatiana F. Vepkhvadze, Evgeny A. Lysenko, Olga L. Vinogradova, Fedor A. Kolpakov, Daniil V. Popov

**Affiliations:** 1 Institute of Biomedical Problems, Russian Academy of Sciences, Moscow, Russia 123007; 2 RIKEN Center for Integrative Medical Sciences, RIKEN, Yokohama, 230-0045 Japan; 3 Institute of Fundamental Medicine and Biology, Kazan (Volga Region) Federal University, Kazan, Russia 420012; 4 Faculty of Fundamental Medicine, M. V. Lomonosov Moscow State University, Moscow, Russia 119991; 5 Institute of Computational Technologies, Siberian Branch of the Russian Academy of Sciences, Novosibirsk, Russia 630090

**Keywords:** Transcription start site, CAGE, Differential TSSs usage, Promoter shift, Transcription factor

## Abstract

**Background:**

More than half of human protein-coding genes have an alternative transcription start site (TSS). We aimed to investigate the contribution of alternative TSSs to the acute-stress-induced transcriptome response in human tissue (skeletal muscle) using the cap analysis of gene expression approach. TSSs were examined at baseline and during recovery after acute stress (a cycling exercise).

**Results:**

We identified 44,680 CAGE TSS clusters (including 3764 first defined) belonging to 12,268 genes and annotated for the first time 290 TSSs belonging to 163 genes. The transcriptome dynamically changes during the first hours after acute stress; the change in the expression of 10% of genes was associated with the activation of alternative TSSs, indicating differential TSSs usage. The majority of the alternative TSSs do not increase proteome complexity suggesting that the function of thousands of alternative TSSs is associated with the fine regulation of mRNA isoform expression from a gene due to the transcription factor-specific activation of various alternative TSSs. We identified individual muscle promoter regions for each TSS using muscle open chromatin data (ATAC-seq and DNase-seq). Then, using the positional weight matrix approach we predicted time course activation of “classic” transcription factors involved in response of skeletal muscle to contractile activity, as well as diversity of less/un-investigated factors.

**Conclusions:**

Transcriptome response induced by acute stress related to activation of the alternative TSSs indicates that differential TSSs usage is an essential mechanism of fine regulation of gene response to stress stimulus. A comprehensive resource of accurate TSSs and individual promoter regions for each TSS in muscle was created. This resource together with the positional weight matrix approach can be used to accurate prediction of TFs in any gene(s) of interest involved in the response to various stimuli, interventions or pathological conditions in human skeletal muscle.

**Supplementary Information:**

The online version contains supplementary material available at 10.1186/s40246-022-00399-8.

## Background

The human genome contains more than 20,000 protein-coding genes. One gene can encode several different protein isoforms, which can have both similar and slightly different functions. The variety of mRNA isoforms is generated and controlled by alternative splicing, alternative transcription start sites (TSSs), and polyadenylation sites. Analysis of RNA sequencing data from mouse nervous tissue at different stages of development [1] and in different human tissues (Genotype-Tissue Expression [GTEx] project database) [2] showed that the alternative TSSs and polyadenylation sites make a key contribution to the expression of alternative mRNA isoforms.

More than half of human protein-coding genes have an alternative TSS [3, 4]. The cap analysis of gene expression (CAGE) approach revealed that in tissues of humans [3, 5] and other organisms [6], as well as during the differentiation of various cells [7, 8] and during embryogenesis in *Drosophila*, zebrafish, and chicken [6, 9, 10], regulation of specific sets of genes is controlled by the appearance/activation of alternative TSSs (differential TSSs usage) within the same or distant promoter region (or nucleosome-depleted region [NDR]). The presence of several TSSs with specific regulatory motifs in a gene plays a key role in tissue- or context-specific regulation of gene expression through various transcription factors (TFs), which might be activated via different signaling events.

It is logical to assume that the change in the transcriptome induced by acute (and transient) stress exposure can also be impacted by differential TSSs usage. Recent studies using the CAGE method have shown the important role of differential TSSs usage in regulating the transcriptome response to acute chemical and physical stress in yeast [11, 12]. To our knowledge, there are no studies in the literature that examine the effects of acute stress on differential TSSs usage in human tissues. Here, we aimed to investigate the contribution of alternative TSSs to the acute-stress-induced transcriptome response in human tissue using the CAGE approach, identifying exact TSS position. Human skeletal muscle is a good model for this task because (i) skeletal muscle is suitable for repeated sampling of muscle tissue with needle biopsies and (ii) skeletal muscle can be easily stressed by exercise (e.g., intense and prolonged [several tens of minutes] aerobic exercise on a bicycle ergometer). During such exercise, mechanical stress acts on muscle cells, intramuscular temperature increases, intramuscular metabolite content changes, and pH and intramuscular glycogen stores decrease. Together, these and other factors activate numerous signaling pathways and markedly change the transcriptome profile (including several hundred genes) of the muscle for several hours during recovery after exercise [13, 14].

The second aim was to create a comprehensive resource of accurate TSSs and individual promoter regions for each TSS in muscle using both ATAC-seq and DNase-seq data—a strong marker of NDRs. This resource together with the positional weight matrix approach was used to identify TFs involved in responses to contractile activity as well as will help to identify TFs involved in responses to other stimuli in human skeletal muscle.

## Results

Ten males exercised intermittently (60 min) on a cycle ergometer (Additional file1: Fig. S1). The pulmonary O_2_ consumption rate (VO_2_) and blood lactate concentration immediately after the high-intensity bouts of intermittent exercise were maintained above 80% of maximal VO_2_ and 5 mM, respectively (Additional file1: Fig. S1) (i.e., the relative intensity of exercise was high, and the exercise induced substantial metabolic stress without progressive metabolite accumulation). Biopsy samples from the *m. vastus lateralis* were taken prior to and at 2 min, 1 h, 3 h, and 6 h after exercise for CAGE (Additional file1: Fig. S1).

### Annotation of CAGE TSSs

**Fig. 1:**
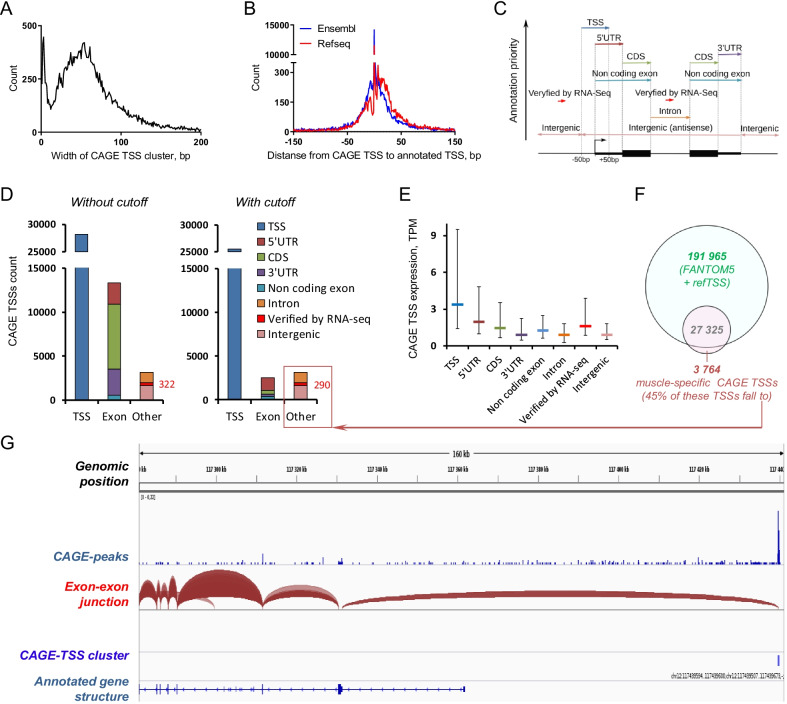
Annotation of cap analysis of gene expression (CAGE) transcription start site (TSS) clusters. **A**. CAGE TSS clusters showed the classical distribution to “sharp” and “broad” classes. **B**. Distribution of distance from CAGE TSS clusters to TSSs annotated in Ensembl and RefSeq (most of our CAGE TSS clusters fall between ± 50 bp from annotated TSSs). **C**. Location of CAGE TSS clusters (ordered by priority) versus gene annotation. Additionally, putative alternative starts were verified using the coverage and exon–exon junction (RNA sequencing data) and annotated to corresponding genes. **D**. Number of CAGE TSS clusters annotated to different locations before (left panel) and after (right panel) elimination of low-abundance CAGE TSS clusters. **E**. Expression (median and interquartile range) of CAGE TSS clusters annotated to different locations. **F**. Overlap of CAGE TSS clusters from the FANTOM5 and refTSS projects with those in our study and 3764 first defined (probably muscle-specific) CAGE TSS clusters. **G**. Analysis of the coverage and exon–exon junctions for the first exon (RNA sequencing data; example for the *NOS1* gene) allows the annotation, for the first time, of 290 CAGE TSS clusters belonging to 163 genes. CDS, coding sequence

In total, we identified 44,680 CAGE-tag-defined TSS clusters (CAGE TSS clusters) (Additional file2: Table S1). In our study, the CAGE TSS clusters showed the classical distribution into “sharp” and “broad” classes of promoters (Fig. 1A), as described previously [3]. To annotate CAGE TSS clusters to genes, we used both the Ensembl and RefSeq annotations. Most of our CAGE TSS clusters fall between ± 50 bp from the annotated TSSs (Fig. 1B). Hence, we used that interval to annotate CAGE TSS clusters as previously annotated TSSs; the annotation priority for other CAGE TSS clusters is shown in Fig. 1C. In total, we annotated 41,951 CAGE TSS clusters to the previously annotated TSSs, exons, and non-coding regions (Additional file2: Table S1). It is noteworthy that elimination of low-abundance CAGE TSS clusters (10% cutoff; see Materials and Methods) substantially reduced (by 30%) the number of annotated CAGE TSS clusters, particularly the number of CAGE TSS clusters annotated to the coding sequence (CDS; by 94%) and 3′ untranslated region (UTR; by 91%) (Fig. 1D; Additional file2: Table S1). These eliminated TSSs belonged predominantly to muscle-specific genes with high expression and/or many exons (e.g., titin, nebulin, and myosin heavy chain 1, 2, and 7) and are probably related to biological and/or technical noise.

The remaining CAGE TSS clusters were compared with the RNA sequencing data from our previous study investigating the effect of aerobic exercise training in human skeletal muscle [15]; verification by coverage and exon–exon junctions for the first exon allowed us to annotate for the first time 290 CAGE TSS clusters belonging to 163 genes (Additional file2: Table S1; Fig. 1D, G). Among them were several well-known protein-coding genes, including nitric oxide synthase 1 (*NOS1*), calcium/calmodulin-dependent protein kinase II alpha (*CAMK2A*), E1A binding protein P300 (*EP300*), ribosomal protein S6 kinase A2 (*RPS6KA2*), homeodomain interacting protein kinase 2 (*HIPK2*), angiomotin (*AMOT*), and homeobox A11 (*HOXA11*), as well as some pseudogenes and long non-coding RNAs. The remaining 2911 CAGE TSS clusters were annotated to introns and intergenic regions (Fig. 1D). The mean expression level of the CAGE TSS clusters annotated to these locations was very low. In contrast, the CAGE TSS clusters verified by RNA sequencing (*n* = 290) show high mean expression level, similar to the CAGE TSS clusters annotated to TSSs and 5’UTRs (Fig. 1E). This finding indirectly confirms the biological significance of the CAGE TSS clusters verified by RNA sequencing in our study.

The CAGE TSS clusters defined in our study overlapped to a large extent with those defined in various human tissues and cells in the FANTOM5 and refTSS projects, which analyzed a limited number of human skeletal muscle samples. This partially explains why we found 3764 new (probably muscle-specific) CAGE TSS clusters (Fig. 1F). However, half of these CAGE TSS clusters were annotated to introns, intergenic regions, and genes verified by RNA sequencing data (Fig. 1F). As mentioned above, only a small fraction of these CAGE TSS clusters showed a high expression level (mainly the CAGE TSS clusters verified by RNA sequencing) (Fig. 1F).

### Alternative TSSs contribute significantly to stress-induced transcriptome response

**Fig. 2:**
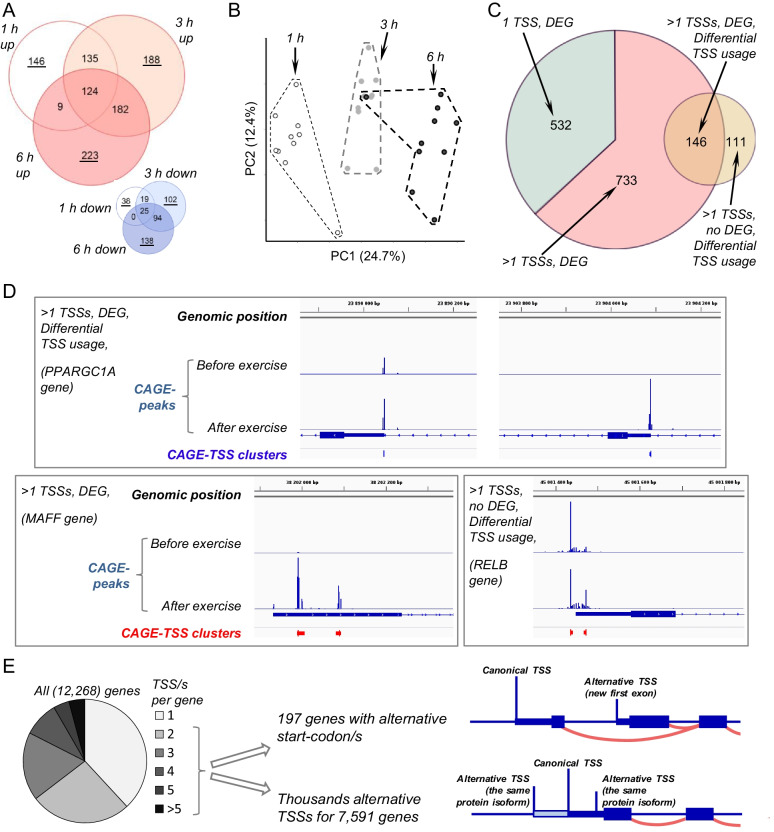
Contribution of alternative TSSs to acute-stress-induced transcriptome response and their functional role. **A**. Number of differentially expressed genes (DEGs) relative to pre-exercise and their overlap at different time points after acute exercise. Up- and down-regulated genes are shown in red and blue, respectively; the number of time-specific DEGs is underlined. **B**. Principal component analysis shows the consistency of gene responses in different volunteers at different time points during the first hours after acute exercise. **C**. Number of DEGs having one (532 genes) or several (733 and 146 genes) TSSs and showing differential TSSs usage (146 genes). Another set of genes (111 genes) shows differential TSSs usage without altering the overall expression of each gene. **D**. Examples of different TSS regulation patterns. **E**. Distribution of TSSs per gene and a potential functional role of the alternative TSSs

Acute exercise changed (mainly upregulated) expression of several hundred genes with a relatively small degree of overlap at each time point (in total 1411 differentially expressed genes [DEGs]) (Fig. 2A, Additional file3: Table S2). Principal component analysis showed that gene responses to exercise at different time points fell into different clusters, confirming the consistency of gene responses in different volunteers (Fig. 2B). Those findings suggest that the transcriptome dynamically changes during the first hours after acute stress. In agreement with previous studies [13, 15], the most highly enriched biological process for upregulated genes in human skeletal muscle was *regulation of transcription* (Additional file1: Fig. S2, Additional file3: Table S2). This is in line with findings in human and mouse cells showing domination of mRNA TFs in the earliest responses to various stimuli [16].

Two-thirds of DEGs have two or more TSSs (alternative CAGE TSS clusters) (Fig. 2C and D), meaning that stress-induced gene expression is related to the regulation of alternative starts. Interestingly, the change in the expression of 146 (~ 10%) of DEGs was associated with the activation of alternative TSSs, indicating differential TSSs usage (Fig. 2C, D, Additional file3: Table S2). Moreover, in another set of 111 genes, differential TSSs usage occurred without altering overall gene expression (the sum of all TSSs related to a gene) and was associated with multidirectional changes in the expression of various TSSs in a gene (Fig. 2C, D, Additional file3: Table S2).

In line with findings in various human tissues [3, 4], we found that 7591 of 12,268 expressed genes have more than one CAGE TSS clusters (Fig. 2E), suggesting that skeletal muscle has a high potential for generating alternative mRNA isoforms. Moreover, 948 of 7591 genes have at least one removed alternative promoter (> 200 bp beyond the promoter region of the canonical TSS—most highly expressed at baseline; see below) of which 89 demonstrate differential TSS usage (Additional file4: Table S3). Importantly, these genes mainly involved in regulation of transcription (Gene Ontology analysis; Additional file3: Table S2), indicating the important contribution of the alternative TSSs belonging to the removed alternative promoters in response to exercise-induced stress. If the alternative TSS is located up- or downstream of the 5′ UTR of the canonical TSS, then this can lead to the appearance of a new first exon(s) and another amino acid residue at the N-terminus (Fig. 2E), which may change the function of the protein. Given the diversity of exon–intron structures of already known mRNA isoforms, predicting all possible mRNA isoforms based on CAGE TSS clusters is a difficult task. Therefore, using data on known mRNA isoforms with defined start codons (Ensembl) and on our CAGE TSS clusters (see Materials and Methods), we found that 197 genes have alternative start codons associated with annotated alternative protein isoforms (Fig. 2E, Additional file4: Table S3). This list can be increased by data from CAGE TSS clusters, which we annotated in our study for the first time (Fig. 1D, G, Additional file2: Table S1). If we assume that most of the alternative mRNA isoforms encoding alternative protein isoforms are already known, then the presence of several TSSs in each of ~ 7000 genes means that the main function of alternative starts is associated not only (to a limited extent) with the generation of alternative protein isoforms but also with the fine regulation of expression of the mRNA isoform from a gene due to the activation of various alternative starts by TFs specific to them (e.g., when the alternative start is located in the 5′ UTR) (Fig. 2E).

### Localization of the individual promoter regions surrounding CAGE TSSs in muscle

**Fig. 3:**
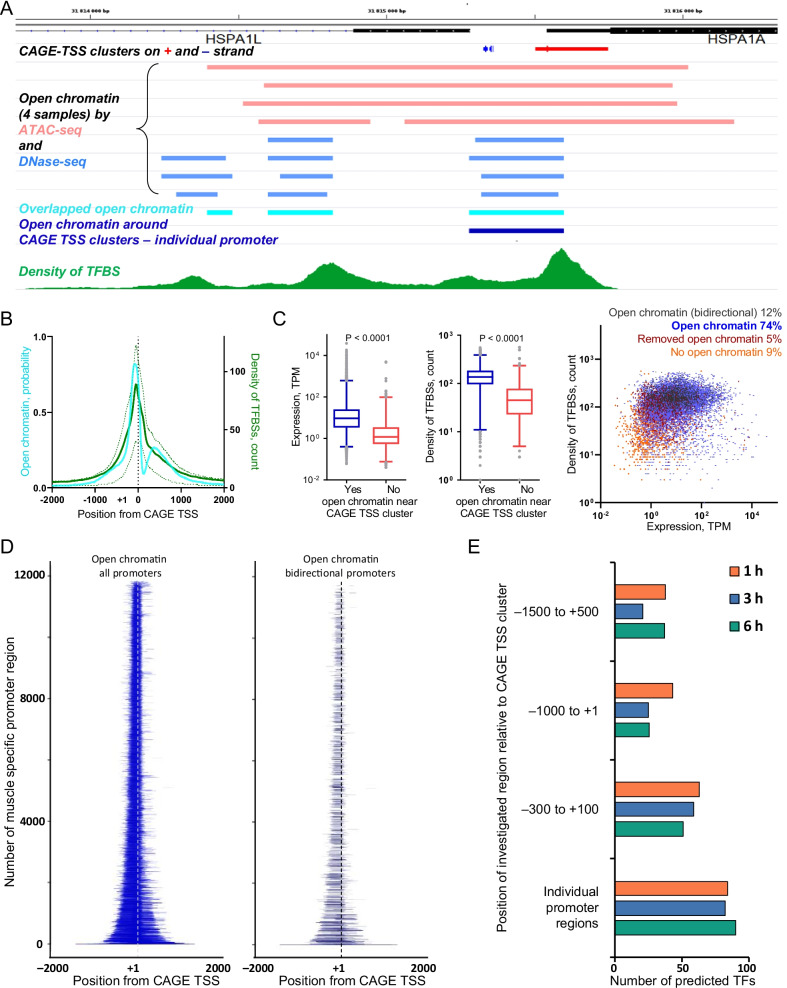
Individual promoter regions for the CAGE TSSs in skeletal muscle. **A**. Individual muscle promoter region around the CAGE TSS cluster was identified using overlapped ATAC-seq and DNase-seq data (MACS2 peaks) (example for a bidirectional promoter); additionally, the density of TFBSs was shown (see Additional file1: Fig. S3). **B**. Open chromatin probability distribution around the CAGE TSS (− 2000 to + 2000 bp) is similar to the density of transcription factor binding sites (TFBSs) (thick and dashed lines are median and interquartile range, respectively). **C**. Individual muscle promoters (86%) show greater expression level and density of TFBSs than a small fraction of pseudo-promoter—a region − 2000 to + 2000 bp from the CAGE TSS clusters without (> 2000 bp from the CAGE TSS cluster) open chromatin. Median, interquartile range, 1–99% range, and *P* value (Wilcoxon test) values are shown. **D**. The individual muscle promoter regions for all promoters and bidirectional promoters are shown (arranged, from bottom to top, according to increasing total length of the promoter; each bidirectional promoter is depicted twice in relation to each TSS). **E**. The use of individual promoter regions increases the number of predicted TFs associated with changes in gene expression at 1 h, 3 h, and 6 h after exercise, compared with the use of standard regions with a fixed length

The positional weight matrix (PWM) method is a classic approach most frequently used for prediction of TFBSs and corresponding TFs responsible for stress-induced DEGs. Estimation of the exact location of each TSS for each gene and the expression level of each TSS is crucial for correct prediction of TFs. Usually, the size of the region around the TSSs in which TFBSs are sought (i.e., a conditional promoter region) is chosen empirically from − 1500– + 500 bp to − 300– + 100 bp (i.e., so-called standard promoter regions). However, the size of promoter regions determined by open chromatin differs significantly for different genes [17, 18]. Therefore, the use of an individual promoter region for each TSS is necessary for the correct prediction of TFs. Because the position of open chromatin can differ between different cells [19, 20], we determined the open chromatin surrounding the CAGE TSS clusters using data from experiments with human gastrocnemius medialis muscle: (see “Methods” section). To identify the individual promoter regions in skeletal muscle, we used the overlap of open chromatin evaluated by ATAC-seq and DNase-seq signals around each CAGE TSS cluster (Fig. 3A). We found an open chromatin probability distribution markedly shifted upstream of the CAGE TSSs (Fig. 3B), as shown previously in yeast [17] and mice macrophages [18].

In addition to open chromatin, a promoter region is characterized by a high density of TFBSs for various TFs [17, 18, 21, 22], which means a high potential for DNA in this region to bind with various TFs. Using data from 15,982 chromatin immunoprecipitation (ChIP)-seq experiments with human cells and tissues (the GTRD database [23]), we determined the density of TFBSs on the DNA around each CAGE TSS (− 2000 to + 2000 bp). Figure 3B shows that the density of TFBSs is similar to the open chromatin probability distribution. Importantly, only a small fraction of the CAGE TSS clusters showed no open chromatin near a CAGE TSS clusters (> 200 bp beyond a CAGE TSS clusters). As expected, these TSSs demonstrate substantially lower expression and TFBS density than those with open chromatin (Fig. 3C).

Because the CAGE TSSs for gene(s) may be located near each other on the same or opposite strand (bidirectional promoters), such CAGE TSSs fall to one promoter region (Fig. 3A). These bidirectional promoters constitute 12% of all promoters and show similar density of TFBSs compared with other promoters with open chromatin (Fig. 3C). As a result of the overlap analysis, we distinguished the coordinates for 11,830 promoter regions in muscle (belonging to 90% of the genes defined in our study) (Fig. 3D, Additional file4: Table S3). No difference in the total length distribution was found between bidirectional and other promoters (Fig. 3D). Then, we identified differentially regulated (exercise-induced) promoter regions in muscle; these promoters demonstrate excellent coincidence with exercise-induced DEGs identified in the study by another bioinformatics approach (Additional file4: Table S3, Fig. 2A).

To examine the ability of the individual promoter regions to better predict TFs than the standard promoter regions, we identified TFs associated with exercise-induced (differentially regulated) promoter for several standard and individual promoter regions by the PWM method (using the TRANSFAC Database v.2020.3 [24] containing matrices for 1357 of 1639 known TFs [25]). The number of predicted TFs with strong adjusted fold enrichment values > 1.5 were then compared. Figure 3E shows that if individual muscle promoter regions are used, the number of predicted TFs is substantially greater than that of standard promoter regions.

### TFs associated with differentially regulated individual promoter regions in muscle

**Fig. 4:**
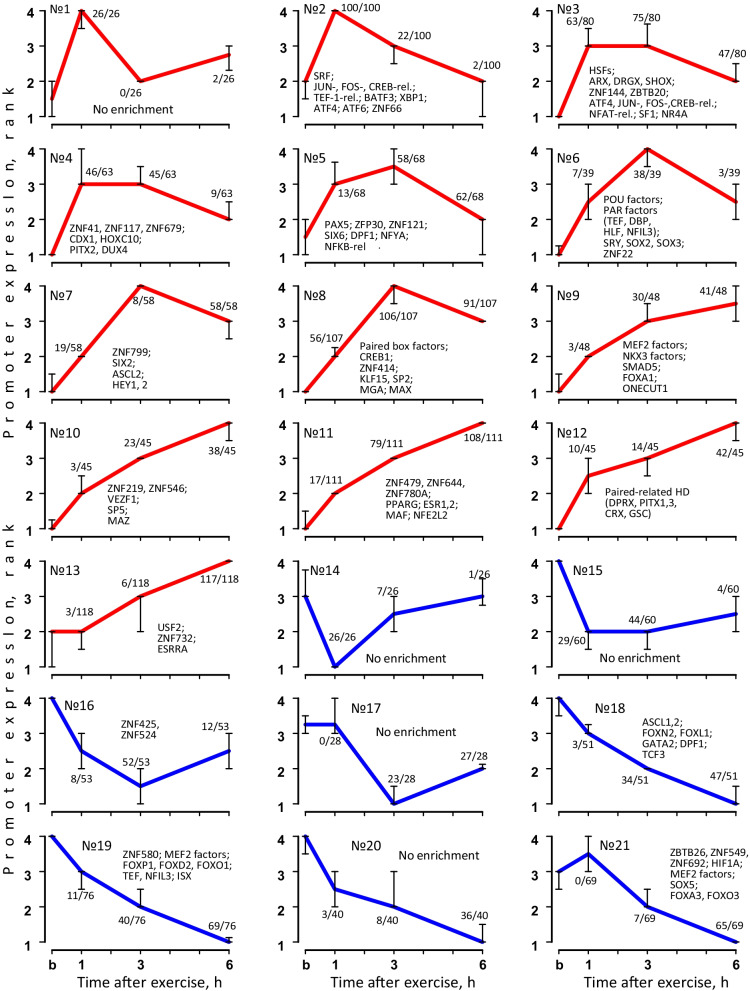
TFs associated with co-expressed individual muscle promoter regions induced by exercise. Unsupervised analysis revealed 21 clusters of co-expressed individual promoter regions induced by exercise (b: before exercise). Each cluster includes data of 10 subjects and 4 time points; expression in each time point for each subject expressed by rank (the highest expression level: 4; the lowest: 1) and is presented as median and interquartile range. Denominator: the number of exercise-regulated individual muscle promoter regions at a time point; Numerator: the cluster size. The top enriched TFs are shown for each cluster

Promoters regulated by a set of TFs should demonstrate similar dynamics of gene expression. Using an unsupervised analysis (the Chinese restaurant process), we identified 21 clusters with co-expressed (presumably co-regulated) differentially regulated individual promoter regions. Then, using the PWM method, TFBSs enriched in the individual promoter regions were predicted in each cluster (Fig. 4, Additional file5: Table S4). Figure 4 shows the average ranked expression of the individual promoter regions and the most enriched TFs for each cluster. Importantly, our approach allows us to identify time points with greater activity of the predicted TFs, meaning time points where the number of exercise-regulated (differentially expressed) individual promoter regions is close to the cluster size (numerator and denominator opposite each point, respectively, in Fig. 4).

The findings are in good agreement with the data in the literature on exercise-induced activation of TFs regulating angiogenesis, mitochondrial biogenesis, and carbohydrate and fat metabolism in skeletal muscle [26]. Namely, nuclear receptors subfamily 4A (cluster 3), as well as estrogen receptors/estrogen-related receptors, peroxisome proliferator-activated receptor gamma and other nuclear receptors (clusters 11 and 13) were found to be associated with increased expression of the individual promoter regions at 3 h and 6 h after exercise, respectively.

On the other hand, our approach allows us to search for TFs (and their potential target genes) with roles in the regulation of stress-induced gene expression in human skeletal muscle that have been studied little or not at all. Thus, early response genes increasing expression at 1 h after exercise (clusters 2–3) were associated with multipotent transcription factors SRF, CREB-, ATF-, FOS-, and JUN-related. An increase in expression at 1–3 h of recovery was associated with calcium-dependent nuclear factor of activated T cells transcription factors and heat shock factors (cluster 3), TFs belonging to the ATF/CREB/AP-1 superfamily (clusters 2, 3, 8), as well as PAX-, NFKB-, POU, PAR-, SIX-, and Krüppel-related factors (KLFs, SPs) (clusters 4–8), while expression at the later stages of recovery was associated with muscle-specific factors MEF2 (cluster 9); NKX3, MAZ, NFE2L2, and paired-related homeodomain factors (cluster 9–13). The zinc finger superfamilies were activated in various combinations during the entire investigated period (clusters 2–8, 10, 11, 13, 16, 19, 21).

## Discussion

In our paper, in contrast to previous studies investigating the alternative TSS usage under near-physiological conditions (in vitro), we examined TSSs in human skeletal muscle at baseline and after acute stress (aerobic exercise). In skeletal muscle, acute exercise (as well other stress stimuli in various tissues or cells) dynamically changes the transcriptome (Fig. 2A, B). We showed, for the first time, that the alternative TSS usage contributes to acute-stress-induced transcriptome response in vivo (Fig. 2C, Additional file3: Table S2). This finding is in line with the response to acute chemical and physical stress obtained in yeast [11, 12], meaning that the alternative TSS usage is a conserved mechanism regulating responses to acute stress. Interestingly, the alternative TSS usage is related to many (77) genes with removed (> 200 b.p.) alternative promoter/s, which highly likely have specific regulatory elements. Those genes include transcription regulators playing the key role in adaptation of skeletal muscle to exercise (*PPARGC1A*, *ESRRG*, *NR4A1*, *NR4A2*, *RARA*, *HDAC5*, etc. [26]; Additional file3: Table S2), indicating the important contribution of alternative TSSs in exercise-induced transcriptome response.

In various human tissues, two-thirds of genes have two or more TSSs ([3, 4] and Fig. 2E), indicating great potential for the expression of alternative protein isoforms. We confirmed about two hundred such examples in human skeletal muscle (Additional file4: Table S3), but the majority of the alternative TSSs do not increase proteome complexity (Fig. 2E). This is consistent with data in other human tissues [2], and with the fact that, in yeast in response to acute stress, alternative starts that change the amino acid sequence are expressed at low levels [12]. Together these findings suggest that the function of alternative TSSs is associated not only (to a limited extent) with the generation of alternative protein isoforms but also mainly with the fine regulation of mRNA isoform expression from a gene due to the TF-specific activation of various alternative TSSs (Fig. 2E). On the other hand, the change of 5’UTR lengths induced by the activation of alternative TSSs could potentially influence mRNA half-life [27] or translation efficiency [28], which play an important role in gene expression regulation.

Using ATAC-seq and DNase-seq data for human skeletal muscle—both strong markers of NDRs—we defined a comprehensive resource of the individual muscle promoter regions for 90% of the genes defined in our study (Fig. 3, Additional file4: Table S3). The lack of the individual muscle promoter regions for remaining genes relates to the lack of open chromatin near the CAGE TSS clusters and may be partially explained by the existence of non-muscle cells in biopsy samples, as well as by the limited number of open chromatin experiments in human skeletal muscle. The use of the positional weight matrix approach together with the individual muscle promoter regions substantially increases the number of predicted TFs compared to standard promoter regions and potentially decreases the rate of false-positive prediction, thereby enabling accurate prediction of TFs in any gene(s) of interest involved in the response to various stimuli in human skeletal muscle. For instance, the combination of this approach and cluster analysis allowed us to predict time course activation of “classic” TFs involved in response of skeletal muscle to endurance like contractile activity [26], as well as diversity of less/un-investigated TFs.

## Conclusions

In conclusion, our work shows that in human skeletal muscle the function of alternative TSSs is associated mainly with the fine regulation of mRNA isoform expression. Transcriptome response induced by acute stress strongly related to activation of the alternative TSSs indicates that differential TSSs usage is a mechanism of fine regulation of gene response to stress stimulus. We created a comprehensive resource of accurate TSSs and individual promoter regions for each TSS in muscle. This resource together with the positional weight matrix approach can be used to accurate prediction of TFs in any gene(s) of interest involved in the response to various stimuli, interventions, or pathological conditions in human skeletal muscle. For instance, the combination of this approach and cluster analysis allowed us to predict time course activation of “classic” TFs involved in response of skeletal muscle to endurance like contractile activity, as well as diversity of less/un-investigated TFs.

## Methods

### Experimental model and subject details

In skeletal muscle of untrained subject transcriptomic response to an aerobic exercise is much greater (and less specific) than that in skeletal muscle adapted to regular exercise [29]. Therefore, amateur endurance-trained athletes [n = 10, median age 32 years (interquartile range, 30–36 years); weight 75 kg (71–78 kg); *V*′O_2max_/kg (maximal pulmonary O_2_ consumption rate) 58 ml/min/kg (54–60 ml/min/kg of body mass)] were involved in our study. An intermittent exercise induces greater molecular response compared to a continuous exercise with the same average power [30, 31]. Hence, each subject carried out an intermittent exercise (60 min, [3 min at intensity 50% of lactate threshold [LT_4_, power at blood lactate 4 mmol/l] + 2 min, 100% LT_4_] × 12) on a cycle ergometer (Ergoselect 200, Ergoline, Germany) 2 h after a standardized breakfast (3582 kJ; 22 g protein, 154 g carbohydrates and 16 g fat). The LT as well as *V*′O_2max_—markers of aerobic performance, were evaluated in a preliminary test session of the incremental cycling test till exhaustion using a Biosen C-line analyzer (EKF Diagnostics, Germany) and a medical gas mass-spectrometer AMIS 2000 with a mixing chamber (Innovision, Denmark). Subjects ate a standardized lunch (3714 kJ; 45 g protein, 183 g carbohydrates and 27 g fat) 1 h 15 min after an intermittent exercise. Biopsy samples were taken under local anesthesia (2 mL 2% lidocaine) using a Bergstrom needle with aspiration from the *m. vastus lateralis* prior to, 2 min, 1 h, 3 h, and 6 h after an intermittent exercise (1st, 2nd, and 3rd from the one leg, 4th and 5th from another leg) (Additional file1: Fig. S1). The muscle samples were quickly blotted with gauze to remove superficial blood, frozen in liquid nitrogen, and stored at − 80 °C until required.

### RNA extraction, library construction, and sequencing

RNA was extracted from the frozen samples (~ 20 mg) using an RNeasy mini kit (Qiagen, Germany) with DNase I treatment (Fermentas, Lithuania); the kit extract RNA with length > 200 bp. The RNA concentration and integrity were evaluated in a fluorometer (Qubit 3.0; Thermo Scientific) and by capillary electrophoresis (Bioanalyzer 2100, Agilent, USA), respectively; all samples were at least 7 in RIN. Libraries were constructed from 2.5 to 3 µg of RNA according to nAnT-iCAGE (non-Amplified non-Tagging Illumina Cap Analysis of Gene Expression) protocol [32] using nAnT-iCAGE Library Preparation kit (DNAform, Japan) and SuperScript III Reverse Transcriptase (Invitrogen, USA). The concentration of the obtained libraries was measured on a Qubit 3.0 fluorometer (Thermo Scientific, USA); the quality of the libraries was checked using an Agilent Bioanalyzer 2100 (Agilent Technologies, USA). The libraries were then validated using real-time PCR (KAPA Library Quantification Kits Illumina, KAPA Biosystems, South Africa) and sequenced on the HiSeq 2000 platform (Illumina, USA) in single-end mode with a length of 50 bp. For each subject samples from time points: prior to, 1 h, 3 h, and 6 h after an exercise were analyzed in one run. Additionally, we analyzed all samples from time point 2 min after an exercise in a separate run. To improve the quality of identification of CAGE TSS clusters, all data were used for calculation. However, differential expressions of CAGE TSS clusters and genes as well as differential TSSs usage were evaluated for time points: prior to, 1 h, 3 h, and 6 h after an exercise only.

Average effective size of the libraries (after depletion of ribosomal and mitochondrial RNAs) was ~ 11 million reads per sample. Raw sequencing data have been deposited to NCBI GEO: GSE164081.

### Data processing and TSSs annotation

Adapter sequences and low-quality reads were trimmed using the Trimmomatic tool (v0.36) (options—illuminaclip:adapters.fa:2:30:10 leading:3 trailing:3 slidingwindow:4:15 minlen:35), then single 5′-guanine was clipped. Genome indexes for alignment were generated by STAR (v2.7.0a) for GRCh38.p13 primary assembly genome (with Gencode annotation v33), then reads were aligned with mismatch rate threshold 0.06. rRNA reads and reads aligned to mitochondrial genome excluded using split_bam.py script (RSEQC v.3.0.1) with combined annotation (RefSeq and Ensembl rRNA genes and mt genome). Only reads with MAPQ = 30 were used for further analysis.

Files with CAGE tags positions (.bed files) were generated by level1.py script (http://genome.gsc.riken.jp/plessy-20150516/PromoterPipeline_20150516.tar.gz) and were used for generation of the CAGE tag-defined TSS clusters (CAGE TSS clusters) by DPI algorithm (https://github.com/hkawaji/dpi1) for all (50) samples. TPM (tags per million reads) expression for the CAGE TSS clusters and counts of CAGE tags per CAGE TSS cluster were estimated by DPI.

Only robust CAGE TSS clusters (TPM at least 1, and count at least 11 for any of the samples) were annotated on the genome (Ensembl (GRCh38.101) and RefSeq (08/15/2020 release)) using R (rtracklayer package) and bedtools v2.26.0 (intersect, closest, sort). To avoid multiple features, an annotation priority of CAGE TSS clusters: TSS (with neighborhood ±50 bp) > 5′UTR > 3′UTR > CDS > exon of non-protein-coding transcripts > intron > intergenic region (Fig. 1C, Additional file2: Table S1) was used. Then, each CAGE TSS cluster was annotated by gene ID. The remained (non-annotated) CAGE TSS clusters were compared with the RNA sequencing data from our previous study (the strand-specific coverage of the first exon and exon–exon junctions, GSE120862, [29]) using Cufflinks-based SEASTAR workflow (https://github.com/Xinglab/SEASTAR). Then, the remaining CAGE TSS clusters were annotated to introns and intergenic regions (Fig. 1C, Additional file2: Table S1).

To reduce the noise, low expressed CAGE TSS clusters (or *minor* CAGE TSS clusters with TPM normalized expression less than 10% of the maximum expressed CAGE TSS cluster for the same gene at any time point) were eliminated. Highly expressed CAGE TSSs were noted as *major* CAGE TSS clusters (Additional file2: Table S1).

### DEGs and differential TSSs usage

Aggregated expression of CAGE TSS clusters per gene (all CAGE TSS clusters annotated to a gene) we used as raw input data for differential expression analysis (DESeq2 R package, analysis of paired samples with the Benjamini–Hochberg correction) with thresholds: |Fold Change|> 1.25 and *P*_adj_ < 0.01. Log_2_ (Fold Change) data were used for the principal component analysis. Genes with differential TSSs usage were identified for each experimental time point using Dirichlet-multinomial-based algorithm [33] from DRIMSeq R package (*P*_adj_ < 0.05).

### Functional gene ontology enrichment

Functional enrichment of protein groups in relation to all detected (~ 11,000) protein-coding genes was performed by the DAVID 6.8 using GOTERM_BP_Direct databases. GO terms with *P*_adj_ < 0.05 (Fisher exact test, Benjamini correction) were regarded as significantly enriched.

### Open chromatin and density of TFBSs around CAGE TSS cluster

To localize genomic intervals with open chromatin around each CAGE TSS cluster (− 2000 to + 2000 bp) in human gastrocnemius medialis muscle (4 samples), we used ATAC-seq (ENCODE data: ENCSR689SDA, ENCSR308HPZ, ENCSR258JCL, ENCSR823ZCR) and DNase-seq (ENCODE data: ENCSR686WJL, ENCSR520BAD, ENCSR791BHE, ENCSR856XLJ) data—MACS2 peaks and normalized signals (See Fig. 3A and Additional file1: Fig. S3). Individual ATAC-seq (as well as DNase-seq) signals were merged; open chromatin positions for ATAC-seq and DNase-seq were overlapped on each other to evaluate general (overlapped) open chromatin intervals (Fig. 3A).

TFBS density around each CAGE TSS (− 2000 to + 2000 bp) was estimated using 15,982 ChIP-seq experiments with human cells and tissues (GTRD database v20.06 http://gtrd20-06.biouml.org/downloads/current/gtrdHub/hg38/bigBed/). Open chromatin probability and average TFBS density were estimated for interval − 2000 to + 2000 bp around the mass center of most expressed TSS cluster in the interval (Fig. 3A).

The calculations were performed using the R environment and bedtools v2.26.0 (merge, intersect, genomecov).

### Individual promoter regions in skeletal muscle and prediction of TFBSs (the PWM method)

The overlapped open chromatin intervals separated by < 71 bp (less than a half of histone wrapped DNA) from each other were joined to an interval. Individual promoter was defined as regions in which open chromatin is located 200 bp or less from the CAGE TSS cluster (Fig. 3A). Other CAGE TSS clusters separated by 200 bp or less were grouped and defined as pseudo-promoters. The pseudo-promoters were divided on promoters with removed open chromatin and without open chromatin. Promoter expression was determined as sum of expression all CAGE TSS clusters related to individual or pseudo-promoter. Maximal TFBS density for each promoter was estimated for each individual promoter interval or pseudo-promoter (− 2000 to + 2000 bp around the most expressed CAGE TSS cluster). The calculations were performed using the R environment and bedtools v2.26.0 (merge, intersect, genomecov).

Differentially regulated individual promoters (as well as pseudo-promoter regions) were determined as described above for DEGs. Enrichment of predicted TFBSs (and corresponding TFs) in individual muscle promoter regions were performed by the geneXplain platform (the “Search for enriched TFBSs (tracks)” function http://wiki.biouml.org/index.php/Search_for_enriched_TFBSs_(tracks)_(analysis)) using the PWM database TRANSFAC v2020.3 [24, 34]. The maximum enrichment (FE_adj_, statistically corrected odds ratios with a confidence interval of 99%) was determined for each PWM (site frequency ≤ 1 per 2000 bp) relative to that in 5000 random individual promoters showing no differential expression in any of experimental time points (DESeq2 method, *P*_adj_ > 0.4). Adjusted fold enrichment (FE_adj_) > 1.5 for transcription factor binding site or promoter’s sequence number (the binomial test and exact Fisher’s test, respectively) and FDR < 0.05 were set as significance thresholds. If a TF has several PWM, the most enriched PWM was used.

### Clusterization of differentially regulated promoters

To search for co-expressed individual promoter regions, ranked expression values were used. Namely, data of four time points (the highest expression—4, the lowest—1) for each person were used for each promoter. The co-expressed promoters were identified by an unsupervised analysis: the Chinese restaurant process (Qin 2006) using the geneXplain platform (function “CRC clustering,” cluster process number = 100, cycles per clustering process = 100, without considering inverted profiles as similar) (http://wiki.biouml.org/index.php/CRC_Analysis). The TFBSs (and corresponding TFs) were predicted using the individual promoter regions by the PWM method (as described above) for each cluster.

### Alternative protein isoforms

The search for alternative protein isoforms was performed for genes having alternative promoter/s. For each individual promoter region or pseudo-promoter region, the most expressed CAGE TSS cluster was determined, which we defined earlier as “TSS” or “5′UTR” (in accordance with Ensembl’s annotation GRCh38.101). Then, the corresponding transcripts and start codons were determined for each CAGE TSS cluster. Finally, genes having at least 2 alternative start codons were identified. The calculations were performed using the R environment, rtracklayer package, and bedtools v2.26.0 (closest).

## Supplementary Information

**Additional file 1. Figure S1**. Design of the study. A. Ten endurance-trained athletes carried out an intermittent exercise on a cycle ergometer. Intensity of exercise was selected based on an incremental cycling test till exhaustion. Biopsy samples were taken from the m. vastus lateralis prior to, 2 min, 1 h, 3 h, and 6 h after an intermittent exercise (1st, 2d, and 3d from the one leg, 4st and 5st from another leg). B. An intermittent exercise induced metabolic shift indicated by markedly elevated blood lactate concentration.** Figure S2**. GO enrichment analysis for biological processes induced by acute exercise. The heat map shows the P-value adjusted (Padj). Dark red and blue denotes the most significant GO terms for up. The count indicates the number of genes enriched into a term.** Figure S3**.Distribution of nucleosome-depleted region markers around the CAGE TSS. The density of transcription factor binding sites (TFBSs) on the DNA (data from 15,982 ChIP-seq experiments; above), mean normalized DNase-seq and ATAC-seq signals (middle) and probability of open chromatin distribution (below) evaluated by normalized signals and MACS2 peaks for DNase-seq and ATAC-seq data, respectively (4 skeletal muscle samples).

**Additional file 2. Table S1**. All and firstly annotated CAGE TSS clusters in the human vastus lateralis muscle. To eliminate low expressed CAGE TSS clusters 10 percent cutoff was applied to CAGE TSS clusters annotated to TSS, 5’UTR, CDS, 3’UTR, Non coding exon, or Verified by RNA sequencing and belonging to a gene. These CAGE TSS clusters were marked as* Major* or* Minor*; no cutoff was applied to CAGE TSS clusters belonging to Intron and Intergenic.

**Additional file 3. Table S2**. DEGs induced in the vastus lateralis muscle by acute exercise and GO enrichment analysis for biological processes.

**Additional file 4. Table S3**. Individual muscle promoters and pseudo promoters. Individual promoter was defined as regions in which open chromatin is located 200 bp or less from the CAGE TSS cluster* Open* – open chromatin near the CAGE TSS cluster. Other CAGE TSS clusters separated by 200 bp or less were grouped and defined as pseudo promoters and divided on promoters with removed (*Closed200*) and without (*Closed2000*) open chromatin.* Closed200* – closed chromatin –200 bp to +200 bp and open chromatin –2,000 bp to –200 bp and/or +200 bp to +2,000 bp;* Closed2000* – closed chromatin –2,000 bp to +2,000 bp). DEP and DEG – differentially expressed promoter and gene, respectively.* Protein isoforms* shows genes having at least 2 alternative start-codons.

**Additional file 5. Table S4**. Clusters of co-expressed individual muscle promoters and TFs associated with the clusters. Clusters shows 21 clusters (C) of coexpressed individual promoters induced by exercise and identified by unsupervised analysis. *Enriched transcription factors* (TFs) show TFs with FDR <0.05 (both the binomial test and exact Fisher’s test) and adjusted fold enrichment >1.5 for both transcription factor binding site (*Adj.site FE*) or promoter’s sequence number (*Adj.seq.FE*).

## Data Availability

All data generated or analyzed during this study are included in this published article and its supplementary information files. All raw and processed sequencing data have been submitted to the NCBI Gene Expression Omnibus (GEO; https://www.ncbi.nlm.nih.gov/geo/) under Accession Number GSE164081. All source codes are deposited in GitHub https://github.com/maxpauel/Differential_TSS_usage_and_acute_stress.
