# Supplementary material for: Alternative transcription start sites contribute to acute-stress-induced transcriptome response in human skeletal muscle
Source: Hum Genomics. 2022 Jul 22;16:24. doi: 10.1186/s40246-022-00399-8 (PMC9308330; doi:10.1186/s40246-022-00399-8)
Supplement: Supplementary file 1 — Additional file 1. Figure S1. Design of the study. A. Ten endurance-trained athletes carried out an intermittent exercise on a cycle ergometer. Intensity of exercise was selected based on an incremental cycling test till exhaustion. Biopsy samples were taken from the m. vastus lateralis prior to, 2 min, 1 h, 3 h, and 6 h after an intermittent exercise (1st, 2d, and 3d from the one leg, 4st and 5st from another leg). B. An intermittent exercise induced metabolic shift indicated by markedly elevated blood lactate concentration. Figure S2. GO enrichment analysis for biological processes induced by acute exercise. The heat map shows the P-value adjusted (Padj). Dark red and blue denotes the most significant GO terms for up. The count indicates the number of genes enriched into a term. Figure S3.Distribution of nucleosome-depleted region markers around the CAGE TSS. The density of transcription factor binding sites (TFBSs) on the DNA (data from 15,982 ChIP-seq experiments; above), mean normalized DNase-seq and ATAC-seq signals (middle) and probability of open chromatin distribution (below) evaluated by normalized signals and MACS2 peaks for DNase-seq and ATAC-seq data, respectively (4 skeletal muscle samples). [file 40246_2022_399_MOESM1_ESM.pdf]

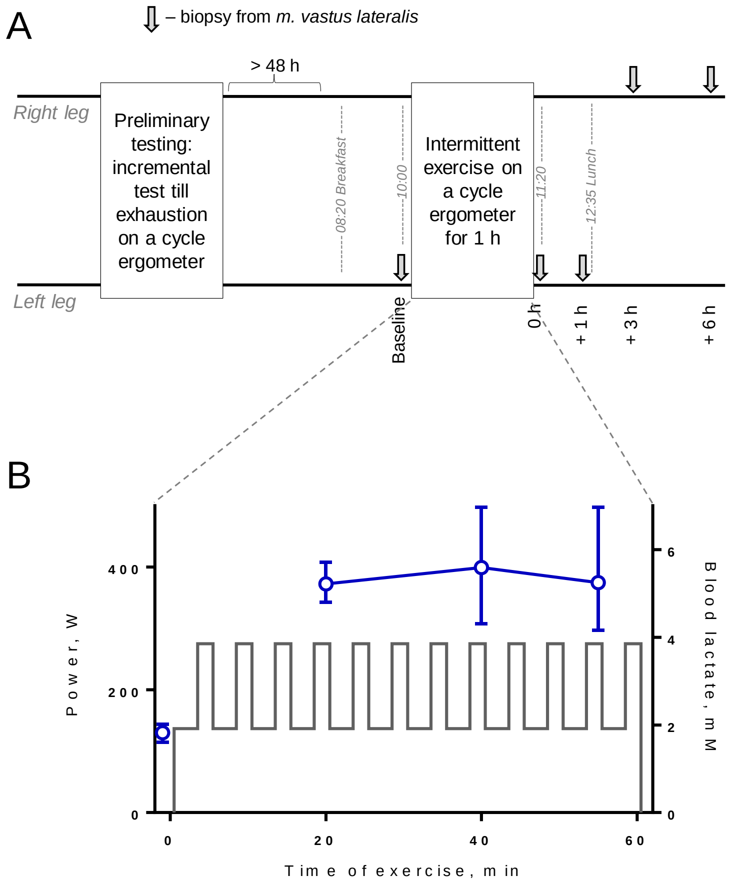

Figure S1. Design of the study

A. Ten endurance-trained athletes carried out an intermittent exercise on a cycle ergometer. Intensity of exercise was selected based on an incremental cycling test till exhaustion. Biopsy samples were taken from the m. vastus lateralis prior to, 2 min, 1 h, 3 h, and 6 h after an intermittent exercise (1st, 2d, and 3d from the one leg, 4st and 5st from another leg). **B.** An intermittent exercise induced metabolic shift indicated by markedly elevated blood lactate concentration.

Common name	GO Biological process	$-log_{10}$ P_{adj} (Count)		
		1 h	3 h	6 h
Regulation of transcription	Negative regulation of transcription from RNA polymerase II promoter	5.56(48)	1.55(49)	2.17(28)
	Positive regulation of transcription from RNA polymerase II promoter	6.31(60)	3.35(69)	
	Negative regulation of apoptotic process	2.34(29)	1.92(36)	
	Positive regulation of gene expression	2.51(21)		
	Negative regulation of sequence-specific DNA binding transcription factor activity	2.11(11)		
	Transcription from RNA polymerase II promoter	1.63(28)		
Regulation of angiogenesis	Positive regulation of cell differentiation	1.63(7)		
	Protein stabilization	1.62(15)		
	Vasculogenesis	1.42(9)		
	Positive regulation of angiogenesis	1.79(12)	3.45(18)	
	Response to unfolded protein	1.61(9)	3.01(13)	
	Platelet aggregation		3.35(11)	
	Positive regulation of cell migration		2.81(22)	
	Leukocyte migration		1.92(16)	
	Positive regulation of cell proliferation		1.92(34)	
	Epithelial to mesenchymal transition		1.42(8)	
	Positive regulation of pathway-restricted SMAD protein phosphorylation		1.42(8)	
Platelet degranulation	Platelet degranulation		3.51(19)	
Response to stimulus	Cellular response to mechanical stimulus		3.18(15)	
	Response to cold		1.42(8)	
Immune response	Immune response	1.67(18)	1.74(23)	
Movement of cell or subcellular component	Movement of cell or subcellular component		1.72(13)	

Figure S2. GO enrichment analysis for biological processes induced by acute exercise

The heat map shows the P-value adjusted (P_{adj}). Dark red and blue denotes the most significant GO terms for up. The count indicates the number of genes enriched into a term.

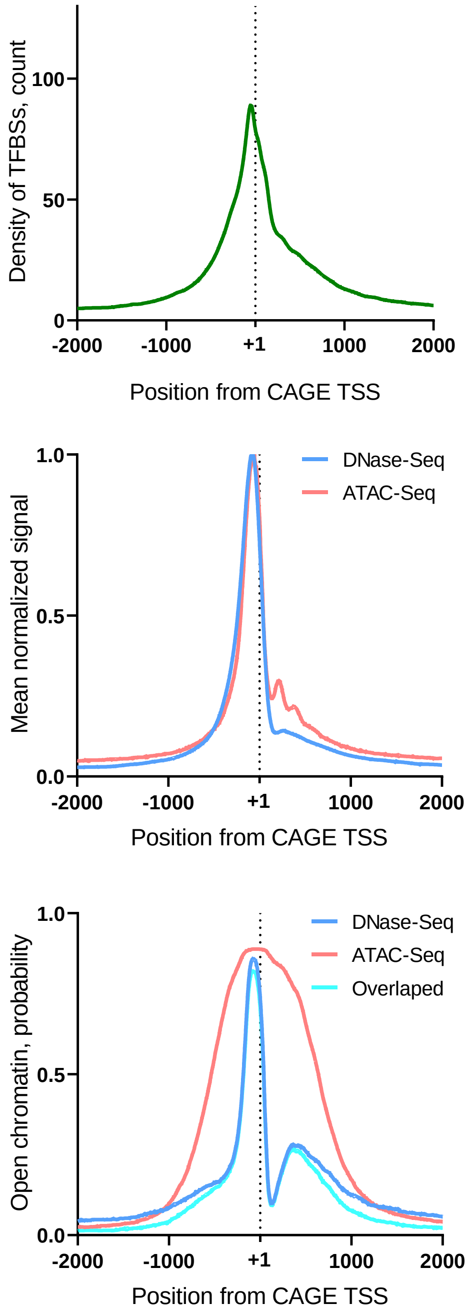

Figure S3. Distribution of nucleosome-depleted region markers around the CAGE TSS

The density of transcription factor binding sites (TFBSs) on the DNA (data from 15,982 ChIP-seq experiments; above), mean normalized DNase-seq and ATAC-seq signals (middle) and probability of open chromatin distribution (below) evaluated by normalized signals and MACS2 peaks for DNase-seq and ATAC-seq data, respectively (4 skeletal muscle samples).
